# Mesenteric cyst in infancy: presentation and management

**DOI:** 10.11604/pamj.2017.26.191.11476

**Published:** 2017-03-31

**Authors:** Samia Belhassen, Braiki Meriem, Laamiri Rachida, Kechiche Nahla, Hidouri Saida, Krichen Imed, Mosbahi Sana, Ksiaa Amine, Sahnoun Lassad, Mekki Mongi, Belguith Mohsen, Nouri Abdellatif

**Affiliations:** 1 University Hospital of Monastir, Department of Pediatric Surgery, Monastir, Tunisia; 2 Research Laboratory LR 12SP13, School of Medicine of Monastir University of Monastir, Tunisia

**Keywords:** Mesenteric, child, surgery, cyst

## Abstract

Mesenteric cysts are documented as a rare entity in pediatric population. They are considered as benign intra-abdominal tumors with an unknown etiology. Symptoms are not specific and knowledge of such condition is essential in order to establish a proper management. We report three pediatrics cases of mesenteric cysts managed between 2000 and 2009 in the pediatric surgery Department of Monastir College Hospital. We described the clinical, radiological and operative findings. Two males and a female were managed (age range: 10 days-5years, mean age: 6,3years). Two patients were presented with an intestinal obstruction. A preoperative diagnosis was made basing on imaging. Thus, abdominal ultrasonography was performed in all of our reported cases and showed a cystic mass in all cases. The cystic nature of the mass, its margins and its extension were better described on tomographic images. The mesenteric cyst was completely and successfully removed in all cases. The histopathological report confirmed the diagnosis and showed a multiloculated cyst with columnar mesothelial lining, without any defined muscular layer or cellular atypia and without any evidence of malignancy. The children were evaluated post-operatively with a mean follow-up of 2 years and a half. No recurrence was noted in our patients during the follow-up period. It is known that clinical features are not specific of such anomaly but once the diagnosis is made, the complete surgical removal of the cyst remains the treatment of choice with excellent outcomes.

## Introduction

Mesenteric cysts are histologically benign tumors. It was demonstrated that their proliferation is caused by lymphatic channels’ obstruction in the mesentery leading to communication rupture with the rest of lymphatic system. Mesenteric cysts are frequently located in the mesentery of the small intestines. Most of them present as symptomless, the preoperative diagnosis is more frequently based on clinical and radiological findings. The histological report confirms the diagnosis. Minimally invasive surgery is the surgical approach of choice. A complete laparoscopic excision of mesenteric and omental cysts is generally feasible. Mesenteric cyst diagnosis pose a challenge as there are no pathognomonic signs and these may mimic pathologies such as cystic lymphangioma and gastrointestinal duplications. In this study, we present completely resectable mesenteric cysts in three case reports, and we specify the clinical presentation as well as the radiological and histological features of these benign tumors.

## Methods

Our experience was based on a retrospective study conducted in the Pediatric Surgery Department of Monastir (Fattouma Bourguiba Teaching Hospital) from 2000 to 2009. Three children were managed. The preoperative diagnosis was primarily based on the clinical features and the radiological assessment. The histopathological report confirmed the diagnosis in all the cases. The children were evaluated post-operatively with a mean follow-up of 2 years and a half.

## Results

### Case report 1

**Figure 1:**
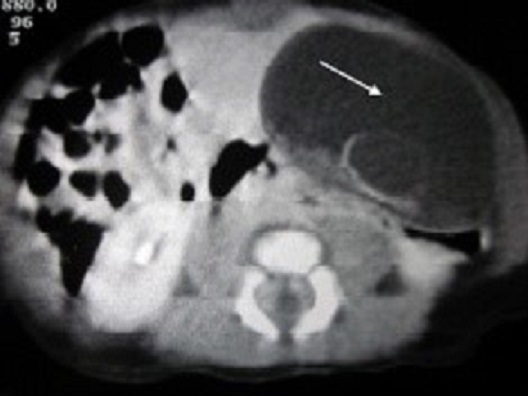
CT scan of the abdomen: voluminous compressing fluid-filled mass with a thin wall and a dense content

A 10-day newborn female baby was admitted for an abdominal cystic mass discovered prenatally. On Physical examination, there was a mass of about 7*5cm at the left lower abdomen. Biological tests were unremarkable. A plain X-ray of the abdomen revealed a radio opaque lesion located at the left iliac fossa which compressed adjacent digestive system. The ultrasonography and the CT scan of the abdomen (Figure 1) confirmed the image. They showed a thin-walled, fluid-filled calcified mass measuring 7cm long axis. The baby was operated using a laparoscopic three-trocar approach. Intraoperatively, the lesion was closely adherent to the sigmoid wall. We performed a complete enucleation of the cyst. The histological report confirmed the diagnosis of calcified mesenteric cyst.

### Case report 2

**Figure 2:**
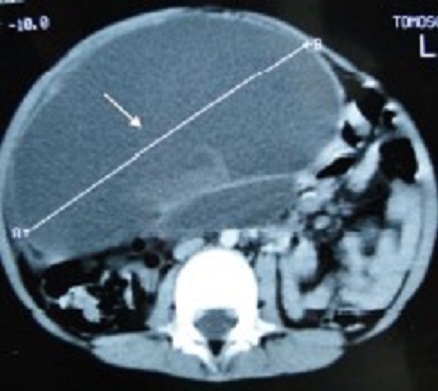
CT scan of abdomen and pelvis showing a large, cystic well defined lesion displacing the bowel anteriorly, it was multiseptated with dense content

**Figure 3:**
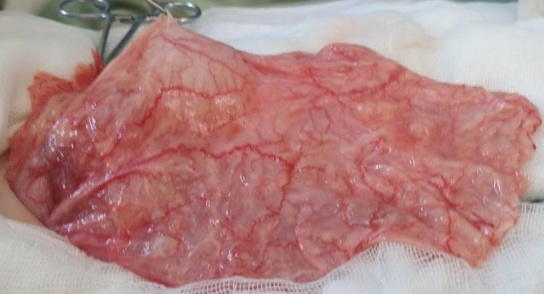
Mesenteric cyst after aspiration of its content, which was a serous fluid

A 4-year-old male child presented with a 2-day history of recurrent abdominal pain, fever and non-passage of both flatus and stools. The physical examination revealed a healthy child with an intra-abdominal voluminous mass measuring 10 cm long axis, on the right upper quadrant with a well-defined margin and smooth surface. The Complete Blood Count was normal. The ultra-sonography and the CT scan of abdomen and pelvis showed a large, cystic well-defined lesion anteriorly displacing the bowel (Figure 2). That mass was multiseptated with dense content; it was located mainly in the abdominal cavity. Those findings were compatible with a mesenteric cyst. The child was surgically managed without delay. During the laparotomy: an intraperitoneal, mutiseptated, large mass was found (Figure 3). It was closely adherent to the digestive wall and originating from the lesser omentum. A complete removal of the cyst was gently performed, thus requiring a segmental resection of the adjacent transverse colon. The child made an uneventful post-operative recovery. The histopathological examination was compatible with a mesenteric cyst.

### Case report 3

**Figure 4:**
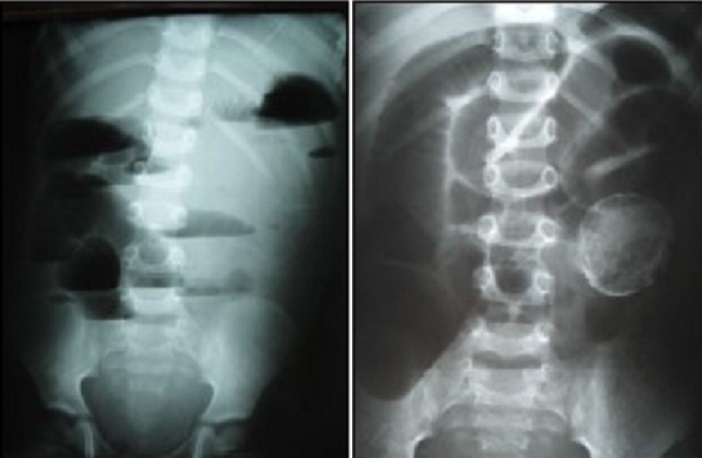
Abdominal plain X-Ray showing multiple air-fluid levels in the small intestine and a radio opaque lesion located at the left lumbar fossa

**Figure 5:**
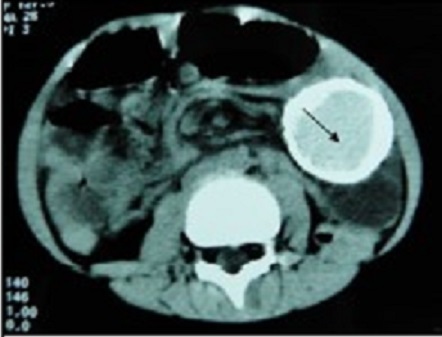
Cystic mass with dense content, enhanced solid portions in its periphery after contrast administration

**Figure 6:**
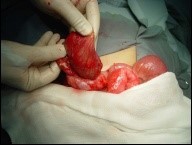
Mesenteric cyst in the small bowel mesentery, closely approximating the bowel wall

A 5-year-old male child was admitted for vomiting, anorexia and weight loss. The routine observations revealed low-grade pyrexia of 37.5%. On physical examination, we noted a palpatory tenderness and a painful mobile mass in the lower left quadrant measuring 4cm long axis. The initial blood tests were all within normal limits. A plain X-Ray abdominal revealed multiple air-fluid levels with a radio opaque lesion located at the left lumbar fossa (Figure 4). We diagnosed that case as an intestinal obstruction. The radiological assessment: the ultrasonography and the computed tomography scan of the abdomen confirmed that image and demonstrated a cystic mass with dense content. Intra-operatively (Figure 5), it was identified as an intestinal volvulus due to a mesenteric cyst originating from the small intestine mesentery and adherent to the bowel wall. That lesion was located at 30 cm proximal to the ileocecal valve on the mesenteric part. A complete excision of the cyst required a small bowel resection (Figure 6). The patient’s post-operative recovery was good. The hospital stay was 9 days. Pathological report of the removed lesion was compatible with a mesenteric cyst.

## Discussion

The first case of mesenteric cyst reported in the literature was by Benevieni in 1507 [1, 2]. Similar pathogeneses are documented in Mesenteric and omental cysts. They are arising from benign multiplication of ectopic lymphatic chanels lacking communication with the remaining normal lymphatic system [3, 4]. Their etiology has not been clearly discovered yet [5]. The mean incidence is estimated at 1 in 100,000 in-patients [4, 6]. These benign tumors are unusual causes of intra-abdominal masses in childhood [7, 8]. The diagnosis is made before the age of 10 years in 25% [7, 9]. The clinical presentation of the lesion depends primarily on the location as well as the size of the cyst. In fact, they can present either with nonspecific abdominal complaints or with acute abdominal pain [7, 8]. The clinical presentation depends also on its associated complications. However many of these cases are asymptomatic and diagnosed incidentally [2]. Abdominal pain is documented as the commonest symptom and an acute abdominal presentation revealing a bowel obstruction is often reported in infants. Rarely an abdominal distension or a mass are found on physical examination [2, 6]. Our experience revealed that two patients presented with an intestinal obstruction. Complications include torsion, infarction, volvulus formation [2, 10, 11] similar to what was observed in our third case report. Other complications were also reported like perforation, infection, anemia from intracystic bleeding, and rupture which is a rare condition usually occuring following an abdominal trauma [4, 12-14]. Cystic abdominal masses are easily evaluated radiologically by ultrasonography, CT scan and MRI [2, 15]. Ultrasound is a very sensitive and specific radiological imaging modality used not only for the diagnosis but also for the follow-up of these cysts, even in the prenatal period [7, 16]. Ultrasonography is a sensitive and specific radiological assessement that provide several radiological features of the lesion. Once an abdominal mass is suspected, ultrasonography should be performed for an initial radiological evaluation. It is feasible and reveals fluid filled cystic lesion. Computed Tomography and Magnetic Resonance Imaging could be helpful and essential in order to obtain a better features description of the mass [4, 17]. Additional informations including lesion origin, its relationships and its adhesion to visceral organs, this can be useful if a laparoscopic surgery is considered [18]. Abdominal ultrasonography was performed in all of our reported cases and showed a cystic mass in all cases. An abdominal computed tomography was carried out and confirmed the cystic nature of the mass, its margins and its extension. The management of this disease is generally performed using a mini-invasive surgery [19, 20]. Laparoscopic approach is well indicated for pediatric population [7]. In this report one patient was managed using laparoscopic approach. Laparotomy was necessary in the remaining 2 cases because of the need for bowel resection and adhesion of the cyst to visceral organs and vessels. The treatment of choice is a total excision of the cyst whenever feasible because of the risk of recurrences. In fact, the sole aspiration of the cyst is not indicated and a complete removal of the cyst is essential and is reported as a procedure of choice to avoid the cyst recurrence as well as its malignant transformation. Segmental bowel resection could be necessary if the intestinal blood supply can’t be preserved especially in huge cysts with excision difficulties [2]. In our report: a complete excision of the cyst required a small bowel resection in two cases: Intraoperatively, the cysts were so huge and closely approximating the bowel wall, therefore a complete excision without intestinal resection was impossible. On histological examination, lesions have an endothelial cell layer, lack muscular lining and are minimally vascular. Once completely removed, cysts have an excellent prognosis with a low risk of recurrence. No recurrence was noted in our patients during the follow-up period.

## Conclusion

As confirmed by our experience, a mesenteric cyst can be diagnosed pre operatively. The knowledge of this rare entity is so important to suspect the diagnosis because there are no specific symptoms. A complete laparoscopic excision of the cyst remains the surgical approach of choice.

### What is known about this topic

- Mesenteric cyst is a rare pathology;
- Symptoms are not specifics;
- Confirmation of this pathology is based on the histological examination.

### What this study adds

- Insists the particularities of this pathology;
- The interest of prenatal diagnosis and screening of this pathology since complications;
- Minimally invasive surgery is the surgical approach of choice to treat and confirm this pathology.
